# Normal and Fibrotic Rat Livers Demonstrate Shear Strain Softening and Compression Stiffening: A Model for Soft Tissue Mechanics

**DOI:** 10.1371/journal.pone.0146588

**Published:** 2016-01-06

**Authors:** Maryna Perepelyuk, LiKang Chin, Xuan Cao, Anne van Oosten, Vivek B. Shenoy, Paul A. Janmey, Rebecca G. Wells

**Affiliations:** 1 Department of Medicine, Perelman School of Medicine, The University of Pennsylvania, Philadelphia, Pennsylvania, United States of America; 2 The Institute for Medicine and Engineering, The University of Pennsylvania, Philadelphia, Pennsylvania, United States of America; 3 Department of Physiology, Perelman School of Medicine, The University of Pennsylvania, Philadelphia, Pennsylvania, United States of America; 4 Clinical Research Center for Diabetes, Tokushima University Hospital, Tokushima, Japan; 5 Department of Materials Science and Engineering, School of Engineering and Applied Sciences, The University of Pennsylvania, Philadelphia, Pennsylvania, United States of America; 6 Department of Bioengineering, School of Engineering and Applied Sciences, The University of Pennsylvania, Philadelphia, Pennsylvania, United States of America; University of California, Berkeley, UNITED STATES

## Abstract

Tissues including liver stiffen and acquire more extracellular matrix with fibrosis. The relationship between matrix content and stiffness, however, is non-linear, and stiffness is only one component of tissue mechanics. The mechanical response of tissues such as liver to physiological stresses is not well described, and models of tissue mechanics are limited. To better understand the mechanics of the normal and fibrotic rat liver, we carried out a series of studies using parallel plate rheometry, measuring the response to compressive, extensional, and shear strains. We found that the shear storage and loss moduli G’ and G” and the apparent Young's moduli measured by uniaxial strain orthogonal to the shear direction increased markedly with both progressive fibrosis and increasing compression, that livers shear strain softened, and that significant increases in shear modulus with compressional stress occurred within a range consistent with increased sinusoidal pressures in liver disease. Proteoglycan content and integrin-matrix interactions were significant determinants of liver mechanics, particularly in compression. We propose a new non-linear constitutive model of the liver. A key feature of this model is that, while it assumes overall liver incompressibility, it takes into account water flow and solid phase compressibility. In sum, we report a detailed study of non-linear liver mechanics under physiological strains in the normal state, early fibrosis, and late fibrosis. We propose a constitutive model that captures compression stiffening, tension softening, and shear softening, and can be understood in terms of the cellular and matrix components of the liver.

## Introduction

Tissues become stiffer in fibrosis and other diseases, including some forms of cancer. This change is used clinically in caring for patients with fibrosis, including liver fibrosis: liver stiffness is viewed as a correlate of fibrosis, and its measurement by transient elastography methods is increasingly used as a diagnostic and predictive tool for fibrosis [1–3]. We have previously shown, however, that increased stiffness precedes as well as results from fibrosis, and that lysyl oxidase-mediated collagen cross-linking can alter liver mechanics independently of matrix deposition [4, 5], suggesting that the relationship between fibrosis and liver mechanics is complex.

Increased tissue stiffness has a direct impact on cell behavior. Most, if not all, cell types respond phenotypically to changes in the mechanics of their environment. Mechanical stimuli have proven to be as important as cytokines, growth factors, and other chemical signals in regulating cell behavior [6–8], and release of active forms of the potent profibrogenic growth factor transforming growth factor (TGF)-β is linked to matrix stiffness [9, 10]. In the liver, cholangiocytes and hepatocytes (the epithelial cells of the liver) are mechanosensitive [11–13], and we have shown that hepatic stellate cells and portal fibroblasts (the myofibroblast precursors of the liver) require a stiff matrix in order to undergo myofibroblastic differentiation [14, 15].

Studying the stiffness of the liver and other tissues has yielded important insights into mechanisms of disease, but stiffness (as quantified by the elastic modulus) is just one component of tissue mechanics. Static stiffness values fail to reflect the full range of tissue mechanics and provide little insight into the contributions of individual tissue components such as cells or the pericellular and extracellular matrix to tissue mechanics. Liver and other tissues are viscoelastic and are composites of cells and matrix polymers, each with unique physical features; their mechanical properties are non-linear, with elastic and viscous properties changing as functions of time and deformation. For example, we and others have shown that liver is strain softening in shear deformation, with the shear modulus decreasing as the strain amplitude increases [5, 16].

Several groups have studied liver mechanics and developed constitutive models to explain liver viscoelasticity. In some cases, this work was designed to model the liver response to high-speed loading (as occurs in blunt trauma such as during automobile accidents); in this setting the liver behaves in some ways similarly to biological polymers such as collagen [17]. In other cases, models were based on shear loading at variable strains and were based on general non-linear viscoelastic models [16, 18–20]. One group has developed a “microchannel flow” model, based on the concept that viscous liquids flow through microchannels in tissues in response to the application of stress, but this makes assumptions of linearity and low strain [21]. A significant issue in developing models of tissue mechanics has been determining how to incorporate tissue heterogeneity–the presence of cells as well as various matrix components with which they interact [22].

Little work has been done on complex liver mechanics in non-trauma settings, such as during the development of fibrosis, although the literature on changes in Young’s modulus in humans with fibrosis is extensive. The extracellular matrix (ECM) of the liver changes qualitatively as well as quantitatively during fibrosis, with increases in fibrillar collagens, which add rigidity, and proteoglycans, which are highly negatively charged and impede fluid flow through the tissue [23]. The mechanical changes associated with these ECM proteins and polysaccharides may contribute to and change cell behavior, especially for myofibroblasts, and have a significant impact on the non-linear mechanics of the tissue. Application of two common viscoelastic models to normal and fibrotic rodent liver (generated by CCl_4_ injection) showed that the stage of fibrosis did not have a linear relationship to either elasticity or viscosity [24], as we have also shown [4].

In addition to changes in resting mechanics that occur in disease, the liver is subject to forces that change significantly in disease, including shear (from altered sinusoidal blood flow) and compression (due to elevated portal pressure in end-stage liver disease). The mechanical response of the liver to these forces is not fully understood but may be relevant to the effective stiffness sensed by cells and therefore to cell behavior. For example, changes in the degree of stiffness sensed by cells (potentially resulting from non-linear responses of tissue to forces like compression) may render the liver more or less susceptible to myofibroblast differentiation and fibrosis. Increased resistance to liver venous outflow (from right-sided heart failure and other causes of chronic passive congestion) leads to liver fibrosis and cirrhosis in humans and experimental animals, underscoring the need to understand the role of forces and deformations on liver mechanics [25–27].

In this study, we systematically evaluated the mechanical properties of the normal and fibrotic rat liver by shear rheometry under a range of shear strains and degrees of axial compression. Perfusion of livers with reagents designed to disrupt cell-matrix contacts, dissolve cell membranes, or disrupt matrix proteoglycans revealed an intimate relation between cells and matrix that produces the mechanical properties of the intact tissue. We suggest the potential relevance of our findings to liver fibrosis and myofibroblast behavior, and propose a constitutive model of the liver that accounts for the non-linear mechanics we observe, including compression stiffening, tension softening, and shear softening.

## Methods

### Animal studies

All animal work was carried out in strict accordance with the recommendations in the Guide for the Care and Use of Laboratory Animals of the National Institutes of Health. Animal protocols were approved by the Institutional Animal Care and Use Committee of the University of Pennsylvania (protocol numbers 804031 and 805338). Animals were housed singly or in pairs in a temperature-controlled environment, with appropriate enrichment, for periods less than several weeks, and with an automatic water system, *ad libitum* feeding of standard rat chow, and 12 h light/dark cycles. Euthanasia was carried out by approved methods (CO_2_ inhalation or sedation with pentobarbital, both followed by exsanguination), and all efforts were made to minimize suffering.

Livers were harvested from 300–350 g Sprague-Dawley rats (Charles River Laboratories, Malvern, PA) and stored in HBSS at 4°C for up to 5 h, then analyzed at room temperature; control experiments indicated that samples are adequately preserved under these conditions and rheological properties are maintained, as reported by others [16]. Perfused livers were analyzed within 1 h of perfusion. Decellularized livers were maintained overnight in PBS at 4°C with shaking. To induce fibrosis, rats on the 2 week CCl_4_ protocol were injected intraperitoneally twice weekly with a mixture of CCl_4_:mineral oil (1:1) at 0.2 ml CCl_4_/100 g body weight x3, and then once with 0.1 ml CCl_4_/100 g body weight. Rats on the 6 week CCl_4_ protocol received twice-weekly intraperitoneal injections of a CCl_4_:olive oil mix (1:1) at 0.2 ml CCl_4_/100 g body weight x4 and then at 0.1 ml CCl_4_/100 g body weight x8. Rats were given DietGel 76A (Portland, ME) during the first two weeks of both protocols to maintain hydration. All tissue harvests were carried out 4 days after the final injection.

### Liver perfusion

Rats were anesthetized with pentobarbital, the abdomens were opened, the portal vein catheterized, and the inferior vena cava transected. Livers were flushed *in situ* with warm HBSS without Ca^2+^ (GIBCO, Manassas, VA), then perfused for 1 h with 400 ml 2% α-amylase (MP Biomedicals, LLC, Solon, OH) in HBSS with Ca^2+^ and Mg^2+^, or for 20 min with 200 ml of 0.025% Triton X-100 in HBSS minus Ca^2+^and Mg^2+^. For treatment with the disintegrin VLO4, which blocks integrin α_5_β_1_, livers were perfused for 30 min with 200 ml of 4.2 μg/ml VLO4 in HBSS with Ca^2+^ and Mg^2+^. VLO4 was isolated from snake venom and generously provided by Cezary Marcinkiewicz (Temple University, Philadelphia, PA) [28]. All treatments were followed by perfusion with HBSS at 37°C for 5 min, then liver harvest. Pump speed was 6.6 ml/min for α-amylase and VLO4 and 10 ml/min for Triton X-100.

### Decellularization

Livers were decellularized *in situ* by detergent perfusion according to a modification of a published protocol [29]. Briefly, after portal vein catheterization, blood was flushed out with PBS and the liver was perfused with 250 ml each of 1%, 2% and 3% Triton X-100 in PBS, 150 ml of 0.1% SDS in PBS, and 250 ml PBS, all at a pump speed of 9 ml/min. After the final perfusion, the livers were removed and washed overnight in PBS.

### Rheometry

**Fig 1:**
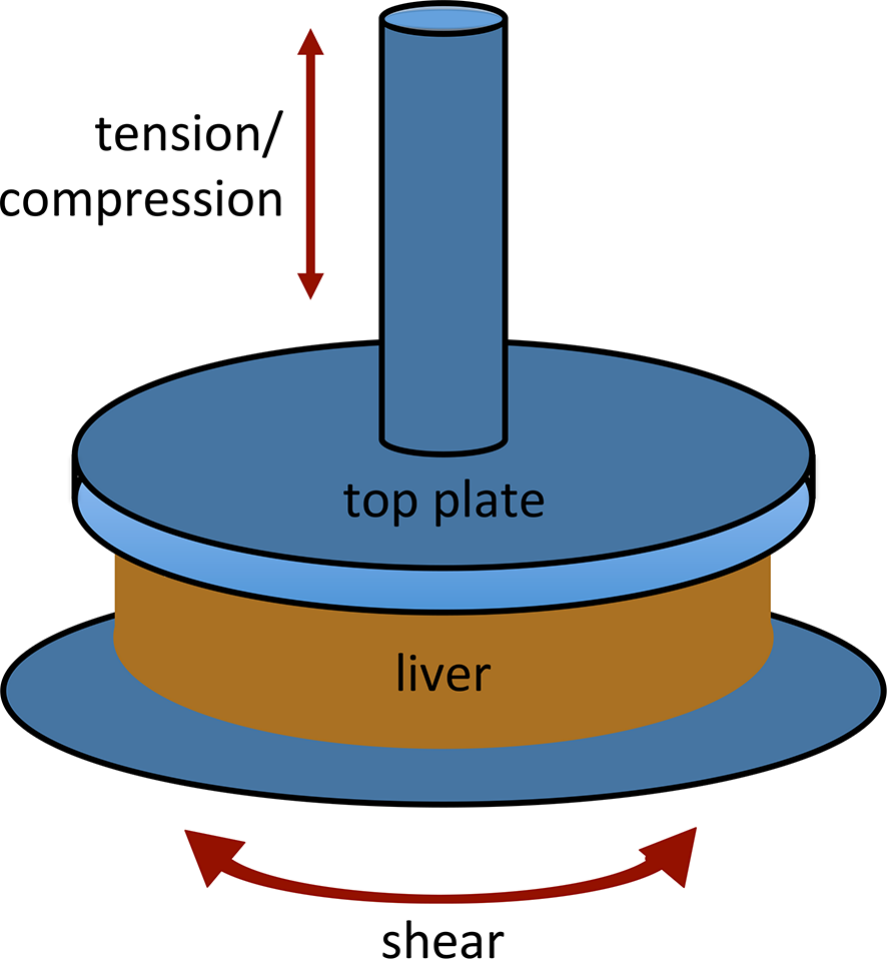
Rheometry methods. Liver samples were cut to a diameter of 20 mm and placed on a parallel plate rheometer with an upper platen of 25 mm. Tension and compression were generated by applying force in a direction perpendicular to the sample. Shear forces were applied by rotating the bottom plate in a direction parallel to the sample. Values derived from compression and tension studies were corrected to account for the difference in size between the sample and the top platen, and for narrowing at the waist of the sample in tension (see methods).

Liver samples were prepared using a 20 mm diameter punch (or an 8 mm punch for poroelasticy experiments) (CL Presser, Philadelphia, PA), with all punches taken in the same orientation. The height of the slices ranged from 3.05 to 5.6 mm in the uncompressed state. Samples were kept hydrated during all experiments with HBSS or (for decellularized livers) PBS. Parallel plate shear rheometry was carried out on an RFS3 rheometer (TA instruments, New Castle, DE) at room temperature using TA Orchestrator software (TA instruments) (Fig 1). Normal forces were simultaneously measured from the analog signal of the RFS3 and collected using Logger Pro software (Vernier Software and Technology, Beaverton, OR).

For all measurements unless otherwise indicated, the upper platen (25 mm diameter) was initially lowered to touch the sample and 10 g of nominal initial force (~300 Pa) was applied to ensure adhesive contact of the sample with the plates. Unless noted, measurements were taken in the following order: dynamic time sweep test (2% constant strain, oscillation frequency 1 radian/s, measurements taken for 120 s, every 20 s); dynamic strain sweep test (increasing strain amplitude from 1 to 50%, measurements taken at 5% increments, oscillation frequency 10 radians/s). Tests were carried out under uncompressed conditions (defined as 10 g initial force) and then with increasing uniaxial compression (10, 15, 20 and 25%), applied by narrowing the gap between the platform and upper platen of the rheometer. The shear storage modulus (G’) was plotted against time (for dynamic time sweep test) or strain (for the strain sweep test), and the shear modulus G was plotted against time (stress relaxation test).

For tension experiments, samples were attached to rheometer platforms with fibrin glue made by mixing equal volumes of 5 mg/ml salmon fibrinogen and 150 U/ml salmon thrombin (Sea Run Holdings, Freeport, ME) immediately before use. 100 μl of the glue was applied to each side of the sample, touching the lower platform and upper platen. 2 g of initial force were applied, the sample was allowed to sit for 5 min to attach to the metal surfaces, and then the force was brought to 10 g. Measurements were taken in the following order: uncompressed (defined as 10 g initial force), 10% tension, and 20% tension. In control experiments, G’ of livers was measured with and without glue and was found to be unchanged, indicating that the glue did not affect the mechanics of the system.

For tension and compression experiments, the following correction was applied to account for the change in cross-sectional area during testing under the assumption that total tissue volume is conserved, where G’ is the storage modulus and λ is axial strain. A similar correction was applied to the loss modulus G”.

$${G\prime }_{actual}={G^{\prime }}_{measured}{\left(1+\lambda \right)}^{2}$$

For reversibility experiments, livers were subject to strain sweeps from 1% to approximately 45% strain. The rheometer was then quickly reset (less than 10 s) and a strain sweep from approximately 50% to 5% was carried out. The rheometer was again quickly reset and the 1% to 45% sweep repeated, to a total of 3 cycles. This was done for 3 individual livers.

### Conversion of normal force into axial stress

Normal force was collected continuously during the duration of the experimental setup (time sweep, strain sweep, at all levels of compression) and was converted to stress by dividing normal force by the area of the sample in contact with the rheometer platens. The correction formula for stress (δ) in tension and compression experiments under the assumption that volume is conserved, was

$$\delta \;actual=\delta \;measured{\left(1+\lambda \right)}^{2}$$

To determine Young’s modulus E, stress (Pa) versus axial strain was plotted, and the slope between two neighboring points was calculated.

### Glycosaminoglycan measurement

Sulfated GAG concentrations were determined using the Blyscan reagent, according to the manufacturer’s instructions (Biocolor, UK). Briefly, small chunks of normal, amylase-perfused, or decellularized liver (up to 50 mg) were placed in 1 ml of digestion buffer (0.2 M sodium phosphate buffer, pH 6.4, 0.1 M sodium acetate, 10 mM EDTA, 0.08% cysteine (Sigma, St. Louis, MO) and 0.02% papain (Worthington, Lakewood, NJ)) and incubated at 65°C for 18 h. The solution was centrifuged at 10,000 x g for 10 min. 30 μl of supernatant and 70 μl of water were combined with 1 ml of the Blyscan dye reagent, incubated for 30 min at room temperature with shaking, and centrifuged at 12,000 x g for 10 min. The resulting pellets were dried and dissolved in 0.5 ml of the dissociation reagent provided. Absorbance was read at 656 nm and concentration determined by comparison to a calibration curve. The average percentage change in liver weight after decellularization was determined for 3 livers per condition and GAG content expressed as a function of starting weight.

### Tissue staining

Samples of all livers were formalin fixed and paraffin embedded, then stained with hematoxylin and eosin or, in some cases, Sirius red and reticulin stains. Laminin and fibronectin immunofluorescence staining was carried out using rabbit polyclonal anti-laminin antibodies (1:100, Abcam, Cambridge, UK) and goat polyclonal anti-fibronectin antibodies (1:100, Santa Cruz Biotechnology, Dallas, TX). Secondary antibodies were from Jackson Immunoresearch Laboratories (West Grove, PA).

### Statistics

In all cases unless stated, data represent the mean ± SD of measurements generated from at least 3 livers. Two-way ANOVA was used to analyze G’ and E data, and unpaired two-tailed t-test for sulfated GAG content.

## Results

In order to understand the mechanics of the normal and fibrotic liver, we carried out a series of studies using a parallel plate rheometer able to simultaneously measure torque and normal force. We applied both compressive and shear strains (Fig 1), aiming for stresses within a physiologically realistic range for a non-traumatic setting, including fibrosis with elevated portal pressures [30] or where there is a tumor or cyst compressing the liver parenchyma (although pressures have not been measured in this setting). Using a rat model, we evaluated normal livers, livers from animals treated with CCl_4_ for two weeks (typical of early fibrosis), and livers from animals treated with CCl_4_ for six weeks (typical of established fibrosis, with bridging and early cirrhosis) (S1 Fig).

### G', G'', and axial stress increase with compression and with progressive fibrosis

**Fig 2:**
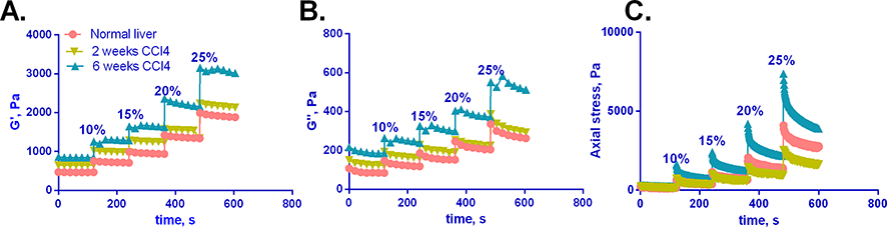
G', G'', and axial stress increase with fibrosis and with compression. (A-C) Normal and fibrotic livers (2 and 6 weeks of CCl_4_ treatment) were subject to varying degrees of axial strain (compression, from 0–25%) as indicated. G' (A), G'' (B) and axial stress (C) were measured over 120 s after each incremental increase in compression. Curves shown are from single livers and are representative of results generated from 3–5 independent livers. (Mean curves +/- SD, representing results from all livers tested, are shown in S2 Fig).

**Fig 3:**
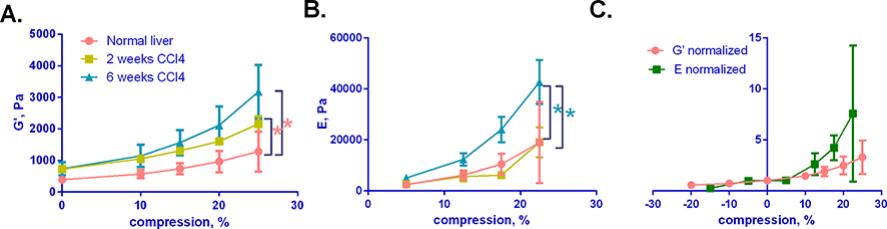
The relationship between G' and E in compression. **(**A) Normal and fibrotic (2 and 6 weeks of CCl_4_) livers underwent shear rheometry with measurement of G’ under varying degrees of compression. G’ shown here was taken at 120 s from the start of compression, approximating equilibrium values after viscous effects have largely dissipated. (B) E was determined by calculating the slope from the stress-strain curve, where stress (calculated from the normal force) was taken at 120 s from the start of compression. The slope was determined in between two neighboring points – 0 and 10, 10 and 15, 15 and 20, 20 and 25 for compression. (C) G' and E for normal livers were calculated as described above in both tension and compression and normalized with respect to initial G’ and E values at no compression. For all cases, 3–5 livers were analyzed for each condition and curves represent the mean +/- SD. By two-way ANOVA, G’ for both 2 and 6 week CCl_4_ livers are significantly higher than for normal livers (p≤ 0.002) pink *; E for 6 week CCl_4_ livers is significantly higher than for normal and 2 weeks CCl_4_ livers (p≤ 0.02) blue *.

We first carried out a time course study, demonstrating increases in the shear storage modulus G', the shear loss modulus G'', and axial stress as fibrosis progresses, as we have reported previously (Fig 2) [5]. Although liver is viscoelastic, G'' is significantly lower than G' (approximately 20%) and contributes little to the complex shear modulus. Notably, liver demonstrates non-linear mechanics, such that G', G'', and E all increase significantly with axial compression (Figs 2 and 3). G’ and E decrease (albeit slightly) with tension (Fig 3C). The increase in G' associated with established fibrosis (6 weeks CCl_4_) is particularly pronounced with compression (Fig 2A).

Normal and fibrotic livers are exposed to a variety of strains, although these have not been studied systematically. Plotting G' as a function of compression shows that compression stiffening is most pronounced at axial strains greater than 15%, and that the greatest divergence between normal, early fibrosis, and established fibrosis also occurs at axial strains greater than 15% (Fig 3A). E shows similar strain dependence (Fig 3B), although it is significantly higher than G' (up to 10 times greater), highlighting that the relation between shear and Young's modulus for liver is not simple as for isotropic continuous elastic materials for which E = 2G(1+ ν), where ν is the Poisson ratio. Interestingly, although both G' and E increase in compression, both change little with increasing tension (Fig 3C). This behavior of liver is in stark contrast to that of collagen gels, which demonstrate increasing G’ in tension and slightly decreasing G’ in compression [31]. Additionally, unlike livers which demonstrate variable E with compression and tension, collagen gels exhibit two distinct E values for compression and tension that are independent of strain: for a 2.5 mg/ml collagen gel, E in tension (~7000 Pa) is approximately 200-fold greater than E in compression (30 Pa) [31].

**Fig 4:**
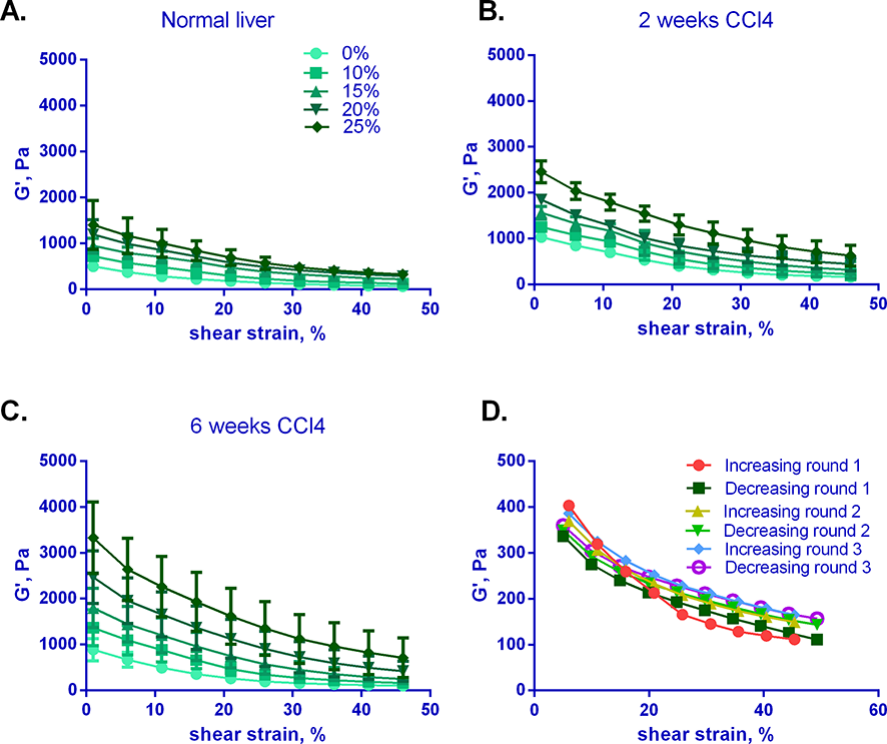
Normal and fibrotic livers demonstrate strain softening and compression stiffening. (A-C) G' was measured for normal and fibrotic (after 2 and 6 weeks of CCl_4_) livers by shear rheometry under increasing strain and under variable degrees of compression ranging from 0–25%. Note that the y-axes scales are the same and that G' values increase significantly as fibrosis progresses. Curves are mean +/- SD for 3–5 livers tested for each condition. (D) Normal liver subjected to three rounds of shear rheometry, each round with increasing, then decreasing strain. Livers demonstrated no evidence of significant tissue damage due to measurements. Representative liver of 3 tested is shown. (See S3 Fig for mean curves +/- SD for all three livers tested.)

We previously reported that normal liver undergoes significant softening with the application of shear strain [5]. We observe shear strain softening even for livers with established fibrosis, although the initial G' at minimal strain is significantly higher for fibrotic livers (Fig 4A–4C). Strain softening persists even in livers under compression, although G' values at all levels of strain are higher under compression, and strain-softening curves are less steep under compression for all livers. Control experiments in which livers were subjected to repeated rounds of increasing and decreasing shear strain indicate that, while there is a slight degree of tissue stiffening after the first round, there is no significant tissue damage with these manipulations, and the mechanical properties of the liver were not sensitive to the history of previous tests (Figs 4D and S3).

### G' increases linearly with compressional stress in normal and fibrotic liver

**Fig 5:**
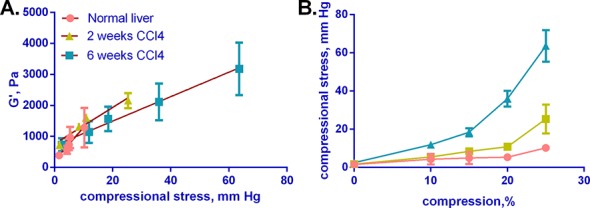
G’ increases linearly with compressional stress, while stress increases nonlinearly with compressional strain for normal and fibrotic liver. Normal and fibrotic (2 and 6 weeks of CCl_4_) livers were subjected to shear rheometry under various degrees of compression. Compressional stress was calculated in mm Hg and plotted against G' or compressional strain (% compression). (A) G' vs. compressional stress, showing a nearly linear relationship between the two conditions in both normal and fibrotic livers. Lines were fit to each curve (in red) and are shown in the graph. (B) Compressional stress vs. compressional strain, shown for normal and fibrotic livers. G’ and compressional stress values are after 120 s of relaxation. Curves reflect mean +/- SD for 3–5 livers per condition.

We then examined the relationships among G', compressional stress, and compressional strain. We observe a nearly linear relationship between G' and compressional stress in normal and fibrotic livers, with similar slopes (Fig 5A); this is particularly interesting because it suggests that uniaxial compression dictates the value of G’. The relationship between compressional strain and compressional stress, however, is nearly linear at low stress (<25 mm Hg) but is non-linear at larger stresses, consistent with the compression-dependent stiffening of the tissues (Fig 5B). The curves for normal and early fibrotic (2 weeks CCl_4_ treatment) liver are similar to each other, but are significantly different from the curve for late fibrotic (6 weeks CCl_4_ treatment) liver. While Fig 5A suggests that equivalent stress at the different compressions yields an equivalent change in G', Fig 5B shows that increased stress is required for deformation in established fibrosis–the same range of stresses results in more strain in early compared to established fibrosis.

Expression of compressional stress in mm Hg suggests that the forces we applied are in the physiologically relevant range (Fig 5B). Excluding consideration of acute changes during trauma, normal and fibrotic livers are subject to significant stresses and strains on a chronic basis. Sinusoidal pressure in a normal liver is less than 10 mm Hg, but may be greater than 20 mm Hg in a cirrhotic liver [30] and the pressure gradient at tissue boundaries will lead to significant strains.

### Poroelasticity contributes little to liver mechanics

**Fig 6:**
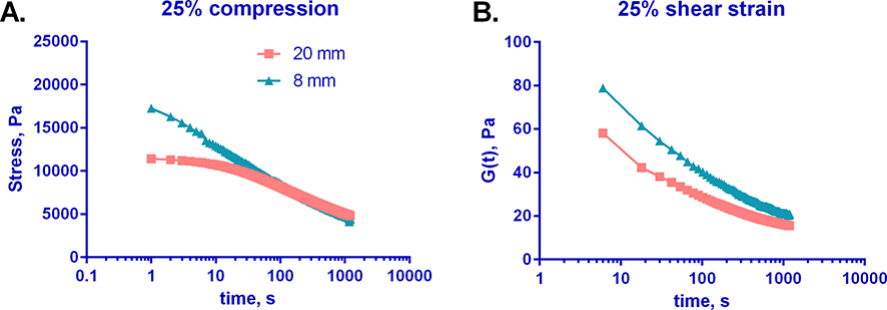
Poroelasticity plays little role in normal liver mechanics. 8 and 20 mm diameter samples were taken from normal liver and subjected to (A) 25% compression or (B) 25% shear strain. Normal force and G' were measured for 1200 s. Stress was subsequently calculated from normal force. There is no statistically significant difference between the curves for the 8 and 20 mm samples for either condition (for the means of 3 samples each) at time points beyond 5 s (which takes into account differences between the initiation of manual compression and the initiation of measurements), suggesting that tissue poroelasticity contributes little to liver mechanics. Curves shown are from a single liver, representative of 3; curves for the remaining 2 livers are shown in S4 Fig.

In order to determine whether poroelasticity plays a major role in liver mechanics on the time scales used for our rheometry measurements, we measured stress and G(t) for normal livers cut to two different diameters and subjected to either 25% compression or 25% shear strain, respectively (Fig 6). In both compression and shear, stress relaxation behavior was effectively independent of sample geometry, suggesting that while poroelasticity of liver is not zero, it contributes little to liver mechanics. (Note that differences in the slope of the curves at time points under 5 s are likely artifacts due to differences in time between manual compression of the samples and initiation of measurements, rather than real differences between the samples.)

### Liver mechanics are influenced by proteoglycans and integrin-matrix interactions

**Fig 7:**
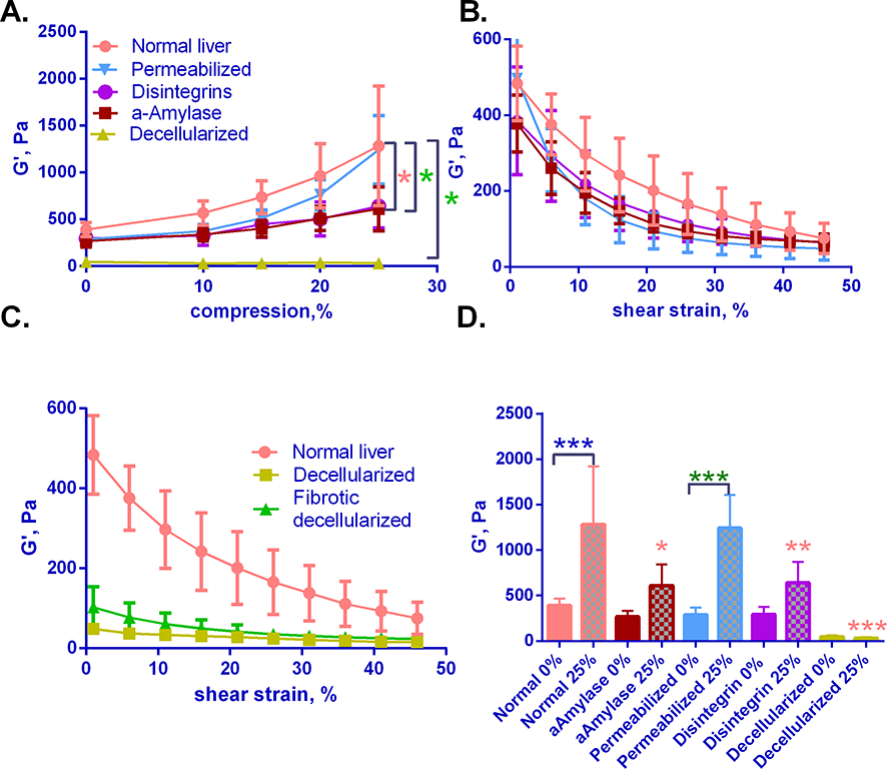
The impact of tissue manipulations on liver mechanics. Normal livers were subject to the manipulations indicated, and then G' was measured under various strains. (A) G' as a function of compression for treated livers. (B) Strain sweeps of normal liver, disintegrin-treated, amylase-treated, and permeabilized livers at 0% compression. Key as in (A). (C) Strain sweeps of decellularized normal and fibrotic (2 weeks CCl_4_) livers at 0% compression, compared to a curve for normal liver (the same as shown in (B)). Note the different shape of the curves for decellularized compared to normal liver. (D) G' measured under 2% oscillatory shear for livers at either 0% or 25% compression. G’ values are after 120 s of relaxation. By two-way ANOVA for the data plotted in (A), G’ for amylase treated livers was significantly reduced from normal and permeabilized livers (brown *, p≤0.02). Disintegrin treatment significantly reduced G’ of normal liver (green *, p = 0) and G’ of decellularized livers was significantly reduced from normal and all other manipulated livers (green *, p = 0). Two-way ANOVA of the data in (D) showed that G’ compared between 0% and 25% compression was significantly different only for normal livers (blue ***, p≤0.001) and permeabilized livers (green ***, p = 0). G’ values at 25% compression were significantly different between normal livers and α-Amylase (*, p≤0.05), VLO4-treated (brown **, p = 0.059), and decellularized livers (brown ***, p = 0). For all graphs, data represent the mean of 3 independent livers +/- SD per condition.

To develop a component-based model of the liver, we perfused normal livers with several reagents designed to disrupt different aspects of liver structure (Fig 7). These included a) α-amylase, which hydrolyzes the internal α-1,4-glucan links in polysaccharides containing 3 or more α-1,4-linked D-glucose units, b) the disintegrin VLO4, which inhibits binding between fibronectin and the integrin α_5_β_1_ [28], and c) varying amounts of detergent to permeabilize or completely decellularize the liver. Control experiments demonstrated that α-amylase and VLO4 perfusion resulted in increased spaces between hepatocytes, although the livers otherwise looked normal (Figures A, C, and E in S1 File). α-amylase treatment resulted in a small but significant decrease in heparan sulfate glycosaminoglycans (Figure F in S1 File). Perfusion with a low concentration of detergent (0.025% Triton X-100) resulted in a slight loss of hepatocytes around the portal regions, but the majority of the liver remained intact (Figure D in S1 File); in contrast, increasing the detergent concentration to 0.1% resulted in extensive cell loss (data not shown). We thus used 0.025% Triton X-100 for permeabilizing liver cells without cell removal. Decellularized livers retained their initial volume, appeared completely white, and demonstrated a complete absence of cells, with preservation of most matrix components except for a significant loss of sulfated GAG residues (S6 Fig), as has been reported by others using similar detergent-based protocols [29, 32–34].

Livers treated in these different ways demonstrated different mechanical properties. With the exception of complete decellularization, all treatments resulted in livers with similar G' values in the uncompressed state at minimal strain (Fig 7A, 7B and 7D). For permeabilization, the data suggest that membrane integrity has a slight effect on overall G', but does not impact compression stiffening; however, measurements related to membrane behavior are probably most accurate at low strains.

Compression stiffening was markedly decreased by perfusion with α-amylase, suggesting that glycosaminoglycans are in part responsible for changes in mechanics with compression (Fig 7A and 7D). Although the total glycosaminoglycan content decreases only slightly (although significantly) by this method, we suspect they are modified after treatment with α-amylase; regardless, it has a marked effect on compression stiffening (Figure F in S1 File). Perfusion with the disintegrin VLO4 also led to marked decreases in compression stiffening, suggesting that links between cells and the fibronectin-rich ECM of the liver also contributed to stiffening with compression (Fig 7A and 7D).

### Decellularization causes marked changes in mechanics

Given the changes observed with cell permeabilization (Fig 7A, 7B and 7D), we determined the effect of complete decellularization on the mechanical properties of the liver. Livers were decellularized by a series of detergent perfusions. Decellularization (which also results in loss of more than 65% of sulfated GAGs; Figure D in S1 File), resulted in an almost complete loss of compression stiffening (Fig 7A and 7D) and in a reduction in baseline G' to approximately 10% that of normal liver. For fibrotic liver (2 weeks CCl_4_), G’ after decellularization similarly decreased to about 10% of baseline. The magnitude of changes after liver decellularization is consistent with that reported for liver by others [35, 36], although it is much greater than for tissues such as lung that are less cellular [37]. In addition to the loss of compression stiffening, decellularization resulted in a marked change in strain softening; at high strains, the decellularized fibrotic and normal livers had the same G' (Fig 7C). This is in contrast to non-decellularized livers, α-amylase-treated livers, and fibrotic livers for which the shape of the strain-softening curves are similar (Figs 7B and 4A–4C). Although we cannot rule out a contribution from GAGs, these data strongly suggest that strain softening is a function of the cellular component of the liver.

### A non-linear constitutive model for the liver

**Fig 8:**
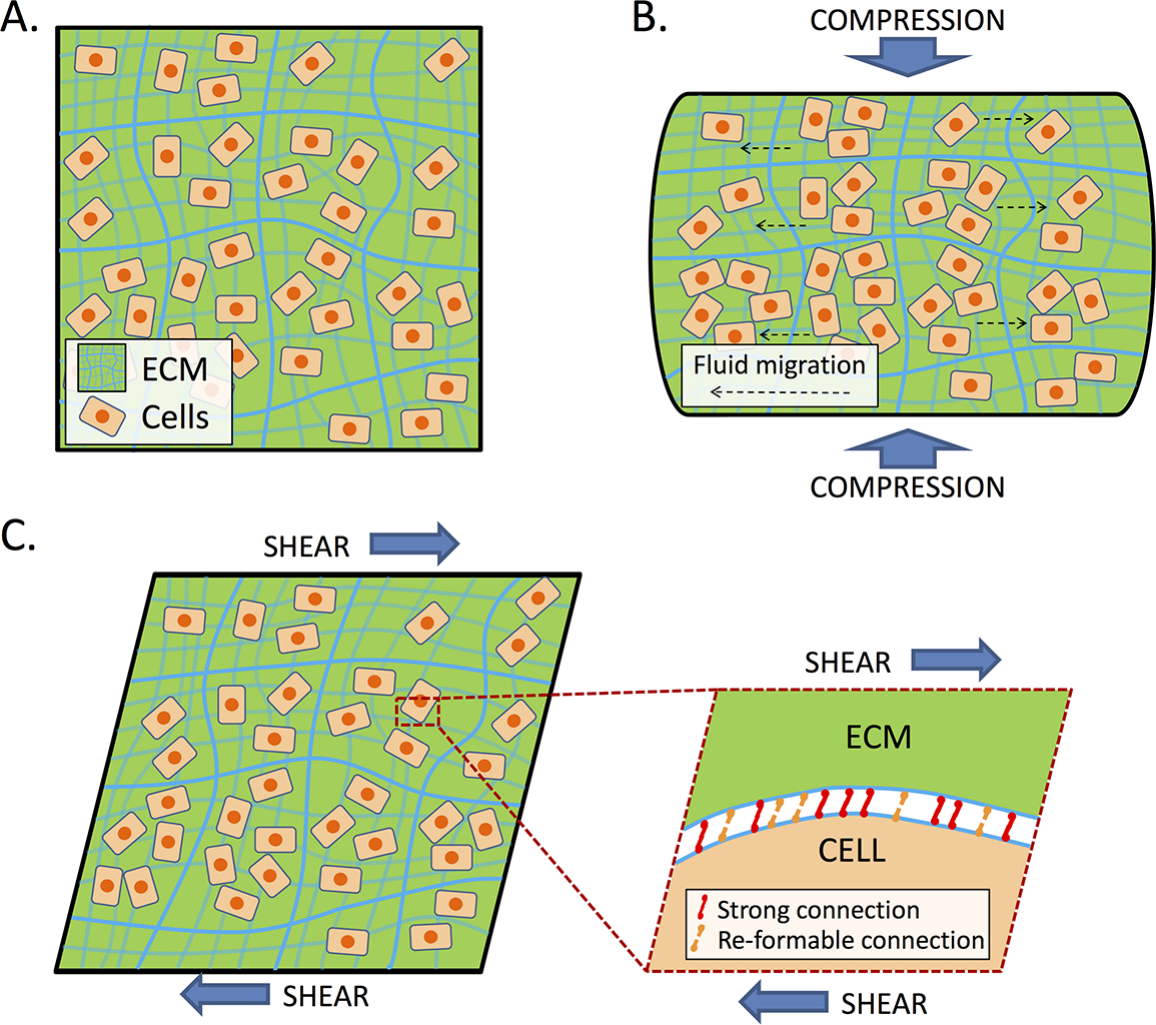
Schematic of the model. A) Schematic for liver tissue, consisting of cells and ECM. B) When under compression, cells maintain the same volume but begin to contact each other and generate strong resistance, resulting in compression stiffening. C) We propose that there are two kinds of cell-ECM connections: strong connections that sustain large loads and re-formable connections that break under large loads and re-form when there is no external load. When under shear, the re-formable connections break, which results in shear softening.

Our data show that both normal and fibrotic liver, unlike gels formed by biopolymers like collagen, demonstrate stiffening in compression and softening in tension and shear. We present a minimal material model to simultaneously capture these behaviors. Derivation of this model is described in S1 File (see Figures A and B in S1 File). A key feature of this model is that, while we assume that the liver is incompressible overall, it contains a high volume of water that can locally flow in and out under mechanical load, and the solid phase can be locally compressible (as is the case for other biological tissues). The material model is characterized by two fitting parameters *C*_1_ and *C*_2_ that can be determined from sheer sweep and compression/tension experiments (Figures C and D in S1 File). To validate the model, predicted values for a) G’ measured at 2% shear strain as a function of axial strain and b) G’ as function of shear strain at different levels of compressive strains are compared to the experimental data (Figures E and F in S1 File). The model predictions are in good agreement with the experimental results, suggesting that the model captures the major elastic response of liver. To the best of our knowledge, this is the first model that captures both compression stiffening/tension softening and shear softening with only two material parameters. A schematic demonstrating the behavior of cells and ECM under compression and shear is shown in Fig 8.

## Discussion

We report here the results of a detailed study of the mechanical properties of normal and fibrotic rat liver as assessed by shear rheometry, with or without uniaxial compression. Unlike tissues and organs such as tendon, heart, and lung, liver has a relatively high cell volume-to-matrix ratio and is relatively soft, making it, like brain and adipose tissue, an interesting system for studying tissue mechanics. Our data corroborate previous findings that liver, like other tissues, demonstrates non-linear mechanics and is viscoelastic [16, 19], although viscosity contributes little to the complex modulus. Although there may be some (non-significant) differences in the stress-time curves of different sized samples, we find that the contribution of *global* poroelasticity to liver mechanics is minimal. The development and progression of fibrosis are associated with increases in G', G'', and E, suggesting that there are changes in the quality of the liver matrix or in tissue reorganization after 6 weeks of fibrosis induction, and that the late fibrotic liver is better able to resist compressive stresses than the early fibrotic or normal liver. Remarkably, we find that both G' and E increase several fold with compression (although they change little with tension), and that G’ and E are not related linearly. These properties cannot be explained by standard models of polymer mechanics and are in contrast to the properties of biopolymers like collagen and fibrin, suggesting that liver mechanics in neither the normal nor the fibrotic liver are a function of matrix content alone. By carrying out a series of manipulations on the liver, we identify the glycosaminoglycan content of the matrix and cell-matrix interactions as key contributors to mechanics, and we propose a novel material model to explain these findings.

We previously demonstrated that liver G' and G'' increase with fibrosis in rat livers [4, 5]. Extensive clinical studies using shear elastography methods to measure E have reached similar conclusions in patients with a variety of fibrotic liver diseases [1, 3, 38]. Interestingly, although increasing stiffness is often assumed to reflect increasing ECM in fibrosis, in neither humans nor animal models does liver ECM content correlate *linearly* with stiffness [3, 4, 39, 40]. Our previous work suggests that increased collagen cross-linking from lysyl oxidase (LOX) family-mediated collagen cross-linking is responsible at least in part for early increases in G' (before the 2 week time point in the CCl_4_ model) in the setting of minimal increases in ECM content [4, 5]. In the current study, we find that stiffness as measured by both G’ and E increases with fibrosis progression, and rises more sharply over the range of physiologically-relevant pressures found in liver disease [30], which is indicative of tissue or matrix reorganization. This may have important implications for cell behavior [14, 15]. Stiffening of the tissue may indicate a protective response to the increased stresses associated with advanced fibrosis. We have not investigated collagen cross-linking at points beyond 2 weeks of CCl_4_ intoxication. Cross-linking occurs relatively slowly (over days to weeks) and others have reported increases in the LOX family member LOXL2 in more advanced fibrosis [41]. It is thus likely that changes in the stress-strain behavior in the 6 week CCl_4_ livers reflect matrix reorganization.

The liver, like brain [42], undergoes marked stiffening in compression [16]. This occurs for normal as well as fibrotic liver, and at stresses which, when converted to mm Hg, are physiologically relevant, consistent with those seen in advanced liver disease. These findings raise the possibility that, in the perfused liver, increases in stress (from increased sinusoidal pressure) result in a liver that is effectively stiffer, and may be sensed as such by cells such as hepatic stellate cells and portal fibroblasts, which undergo stiffness-dependent differentiation into fibrogenic myofibroblasts [14, 15]. This may explain the increased stiffness observed with *in vivo* compared to *ex vivo* measurements. Our measurements were all carried out *ex vivo*, but it would be interesting to carry out shear rheometry with compression in perfused livers. Both normal and fibrotic livers demonstrate shear strain softening as well as compression stiffening, and we observe a slight decrease in G' (and E) with tension, as has also been observed for brain [42]. Given that livers are not fixed in place but may be subject to a variety of multiaxial external and internal strains or forces, stiffness in the liver *in vivo* may be quite variable.

Perfusion with α-amylase, which removes a small fraction of glycosaminoglycans causes a decrease in the degree of compression stiffening, suggesting that glycosaminoglycans are at least partly responsible. Glycosaminoglycans carry densely packed negative charges and as a result there may be significant osmotic shifts associated with compression, leading to stiffening. Decellularized livers, which demonstrate marked loss of glycosaminoglycans, show an almost complete loss of compression stiffening, which is consistent with a role for glycosaminoglycans, although there are multiple additional changes in these livers, including loss of cell-matrix adhesions, which could also affect their mechanical properties. Perfusion with VLO4, which decreases cell-matrix adhesions, also causes a decrease in compression stiffening. Consistent with this observation, there are published data suggesting that glycosaminoglycans and cell-ECM adhesions are linked [43].

The mechanical behavior of the liver (as well as brain [42] and adipose tissue bears some similarity to networks of biopolymers such as collagen. Like these networks, E and G do not follow the relationship E = 3G (Fig 3) typical of linearly elastic materials. Comparing the shear modulus with uniaxial compression of gels formed by biopolymers such as collagen and fibrin with that of tissues, however, shows a striking divergence between the mechanical behavior of the liver and of biopolymers. Reconstituted fibrin and collagen networks compression soften, and strain stiffen, the opposite of liver tissue [31]; this makes it clear that soft tissue mechanics cannot simply be modeled on or explained by collagen gels. Even if increased collagen crosslinking or collagen expression leads to increased liver stiffness *in vivo*, the magnitude and strain-dependence of the stiffening effect need not arise directly from the increased collagen network stiffness but might instead involve the cellular response to the change in matrix stiffness. Our findings showing that fibrotic rat livers even after 6 weeks of CCl_4_ treatment (when they typically demonstrate early cirrhosis) demonstrate compression stiffening and strain softening highlight that the mechanical changes in fibrosis cannot simply be modeled as increased collagen content.

Rather than modeling the liver as a complex system of polymers with only elastic and viscous constants, our experimental results suggest that the mechanical behavior of the liver largely arises from a tight coupling of cells to the matrix, as demonstrated by interruption of cell-matrix polymer interactions through decellularization or disintegrin treatment. Decellularization, which completely removes all cells, leads to a dramatic reduction in G' and effective loss of both compression stiffening and shear strain softening, but does not restore strain-stiffening to the remaining material as would be expected for a network of collagen (Fig 7A, 7C and 7D). Cell permeabilization, which should not affect the cytoskeleton or cell-matrix interactions, has minimal effect on compression stiffening or shear strain softening, particularly at high compression or shear, while perfusion with the disintegrin VLO4, which disrupts the linkage between integrin α_5_β_1_ and fibronectin, causes a decrease in compression stiffening. This is consistent with the model that cells and their linkages to the matrix are important participants in tissue mechanics. The liver is approximately 85% cellular by volume [44]. Although we have not investigated tissues like heart or kidney, we would predict, given their increased matrix-to-cell volume ratio compared to liver, that they would demonstrate less extreme shear strain softening and compression stiffening (as well as decreased changes after decellularization), although the trends would remain the same. It will be important to study livers with end-stage cirrhosis, where the matrix-to-cell volume ratio is markedly different than in the early cirrhotic (6 weeks of CCl_4_) livers we investigated.

In developing a new constitutive model to capture the non-linear properties of the liver as a representative tissue, we considered that the ECM is a network of collagen and other ECM proteins, which can considered to be fibrous material permitting fluid to flow freely in and out. When the tissue is under compression, fluid in the ECM flows out and cells begin to contact each other. Since fluid, especially water, is not easy to transport across cell membranes, contact between cells generates significant resistance and therefore results in compression stiffening. The connections between cells and the ECM in tissue can be divided into two groups: strong connections that can sustain large loads without breaking and re-formable connections that are easy to break when the load increases beyond a threshold level (integrin affinity can be regulated by allosteric interactions or force). Broken re-formable connections can re-form when the load is dismissed. When the tissue is under shear, the re-formable connections begin to break and therefore lead to shear softening. When the load is dismissed, the strong connections ensure that the tissue resumes its original state and the re-formable connections begin to re-form. Clearly, since cells play an essential role in both mechanisms, compression stiffening and shear softening are not expected when cells are removed from the tissue. This is consistent with the experimental observation that decellularized liver displays almost no compression stiffening or shear softening when compared with normal liver, and also highlights the key role of cell-matrix interactions in determining liver mechanics.

We believe that our findings with rat livers are relevant to human livers. Administration of CCl_4_ is a common method for inducing fibrosis in rodent models. It is a reproducible and recapitulates the pathology and stages of human fibrosis–inflammation, regeneration, matrix deposition, and regression–although it is highly toxic [45]. With respect to material properties, human liver may be slightly stiffer than rat, and mouse liver slightly softer, as determined by preliminary rheological measurements in our laboratory. Both human and mouse livers also exhibit the compression stiffening and strain softening reported here for rat livers. Clinically, human liver stiffness as measured by ultrasound elastography [46, 47] is higher than the uncompressed stiffness values for rat liver we report, but is similar when livers are under physiologically-relevant compression levels. Rat livers and the CCl_4_ rat fibrosis model thus appear to be suitable models for human livers with respect to pathology and mechanics, although further investigation is needed and is of ongoing interest in our laboratory.

In sum, we report a detailed experimental and theoretical study of liver mechanics. We propose that both cells and the matrix are significant contributors to the mechanical properties of the tissue, and that cell-cell and cell-matrix contacts largely drive these properties. These mechanical properties have significant implications for the behavior of cells within the tissue, suggesting that the effective stiffness sensed by cells may be highly variable, and may regulate progression of fibrotic disease.

## Supporting Information

S1 Fig Histology of rat livers. For all experiments, rat livers were used after (A) no treatment (normal), (B) 2 weeks of CCl_4_ intoxication (early fibrosis), or (C) 6 weeks of CCl_4_ intoxication (established fibrosis). Samples are representative of 3–5 livers per condition, and were stained with Sirius red, which detects collagen. Magnification 100X.
(JPG)

S2 Fig G', G'', and axial stress increase with fibrosis and with compression. **(**A-C) Normal and fibrotic livers (2 and 6 weeks of CCl_4_ treatment) were subject to varying degrees of axial strain (compression, from 0–25%) as indicated, and G' (A), G'' (B), and axial stress (C) were measured for 120 seconds for each incremental increase in compression. Curves shown represent means of data from 3–5 independent livers. Representative curves from single livers are shown in Fig 2, for clarity. Curves in all panels are mean +/- SD.
(TIF)

S3 Fig Normal liver subjected to three rounds of shear rheometry, each round with increasing, then decreasing strain. There was no evidence of significant tissue damage due of significant tissue damage due to measurements. Means ± SD of 3 tested livers are shown. A set of curves from a single representative liver is shown in Fig 4D for clarity.
(TIF)

S4 Fig Response of normal liver to 25% compression and shear strain. Panels (A) and (B) show the remaining two individual livers from the experiment described in Fig 6. 20–1 represents the first liver, cut with a 20 mm punch, while 8–1 represents the same liver cut with an 8 mm punch; 20–2 is a second liver cut with a 20 mm punch, and 8–2 the same liver cut with an 8 mm punch. The data in Fig 6 are from a third liver.
(TIF)

S5 Fig Hematoxylin and eosin stains of fixed livers. (A-E) H&E-stained fixed livers: (A) normal, (B) fibrotic (2 weeks CCl_4_), (C) perfused with 2% α-amylase, (D) permeabilized with 0.025% Triton X-100, and (E) perfused with the disintegrin VLO4. F. Blyscan assay results showing mean +/- SD of the relative heparan sulfate proteoglycan content of normal *vs*. α-amylase-perfused livers. Note that spaces between hepatocytes appear larger in α-amylase- (C) and disintegrin-perfused (E) livers. There is mild loss of periportal cells in 0.025% Triton X-100 perfused livers, but the architecture is maintained overall. Magnification is 200X for all pictures, and all are representative of a minimum of 3 livers examined. (F) Represents analysis of three livers per condition, mean normalized to 100% for the unperfused, +/- SD, p<0.05.
(TIF)

S6 Fig Characterization of decellularized livers. Livers were perfused *in situ* with increasing concentrations of Triton X-100 followed by 0.1% SDS (all in PBS), and were then washed with PBS. (A) Hematoxylin and eosin staining of normal decellularized liver. (B) Hematoxylin and eosin staining of liver treated for 2 weeks with CCl_4_ then decellularized. (C) Appearance of a decellularized normal liver. The size of the liver was maintained after perfusion. (D) Sulfated glycosaminoglycan (sGAG) content. Liver samples were analyzed with the Blyscan reagent. Livers decellularized by detergent perfusion retain on average 34% of initial GAGs. Mean +/- SD (normalized to 100% for control livers) of data from 3 individual livers. ***, p<0.0001 by t-test. (E-L) Paraffin sections of intact (E-H) and decellularized (I-L) normal livers stained with Sirius red (detects overall collagen; (E,I) and reticulin stains (collagen type III and proteoglycans; F, J) and with antibodies against laminin (G, K) and fibronectin (H, L). Collagen fibrils appear yellow when stained with sirius red and visualized with polarized light (E, I), and black when reticulin stained (F, J); for (G, H, K, L), specific stain is red, DAPI blue. Note the absence of specific staining for nuclei in the decellularized livers. (E, I) 40X; all others, 200X.
(TIF)

S1 File A non-linear constitutive model for the liver. (A) Sample dimension parameters. (B) Predicted stress vs. strain λ (left) and modulus vs. strain λ (right), both normalized to *C*_1_. (C) Relation between G’ and shear strain γ. G’ decreases with application of shear strain. (D) Relation between E and axial strain λ. E increases with application of compressive strains and decreases with tension. (E) The model predictions for the relationship between G’ and axial strain λ. G’ increases with increasing compression and decreases with increasing tension, which is comparable to experimental data. (F) The model predictions compared with experimental data for the relationship between G’ and shear strain γ at different levels of compressive strains. G’ decreases with increasing shear at all levels of compression.
(DOCX)
