# Supplementary figures and images for: Normal and Fibrotic Rat Livers Demonstrate Shear Strain Softening and Compression Stiffening: A Model for Soft Tissue Mechanics
Source: PLoS One. 2016 Jan 6;11(1):e0146588. doi: 10.1371/journal.pone.0146588 (PMC4703410; doi:10.1371/journal.pone.0146588)

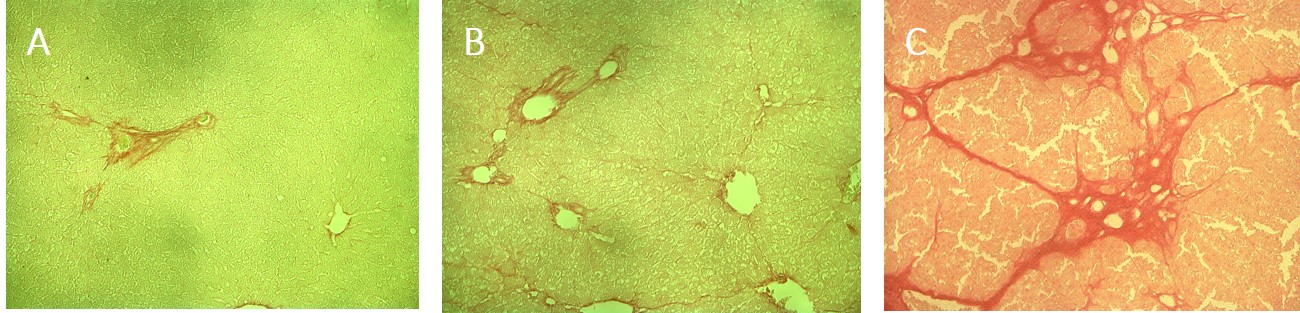

Supplement: S1 Fig — For all experiments, rat livers were used after (A) no treatment (normal), (B) 2 weeks of CCl4 intoxication (early fibrosis), or (C) 6 weeks of CCl4 intoxication (established fibrosis). Samples are representative of 3–5 livers per condition, and were stained with Sirius red, which detects collagen. Magnification 100X. (JPG) [file pone.0146588.s001.jpg]

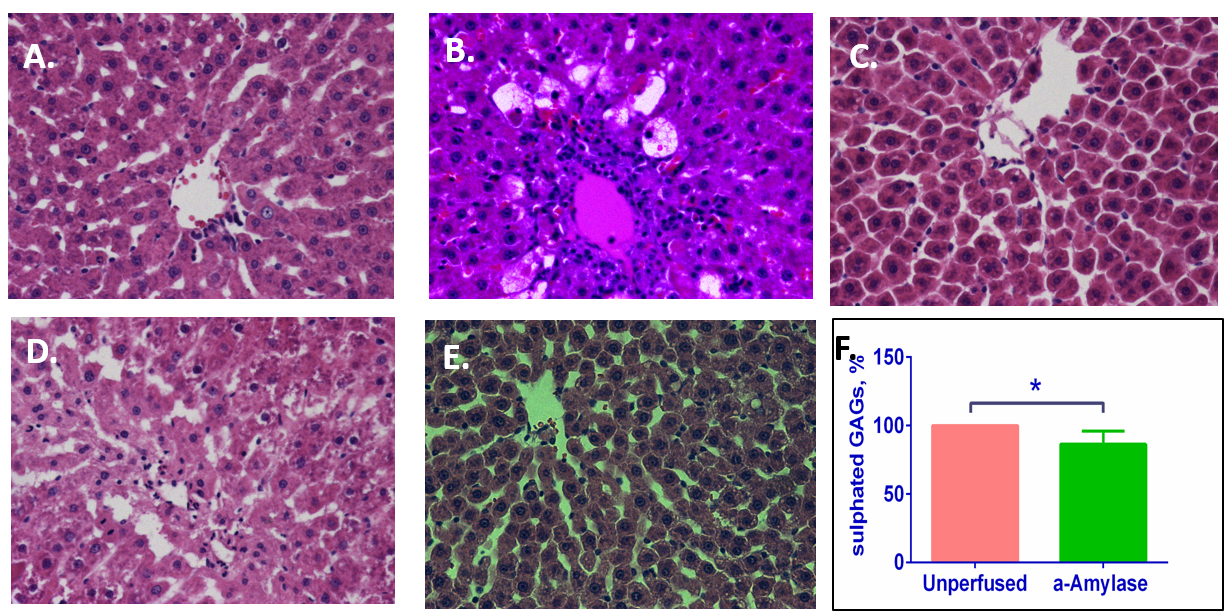

Supplement: S5 Fig — (A-E) H&E-stained fixed livers: (A) normal, (B) fibrotic (2 weeks CCl4), (C) perfused with 2% α-amylase, (D) permeabilized with 0.025% Triton X-100, and (E) perfused with the disintegrin VLO4. F. Blyscan assay results showing mean +/- SD of the relative heparan sulfate proteoglycan content of normal vs. α-amylase-perfused livers. Note that spaces between hepatocytes appear larger in α-amylase- (C) and disintegrin-perfused (E) livers. There is mild loss of periportal cells in 0.025% Triton X-100 perfused livers, but the architecture is maintained overall. Magnification is 200X for all pictures, and all are representative of a minimum of 3 livers examined. (F) Represents analysis of three livers per condition, mean normalized to 100% for the unperfused, +/- SD, p<0.05. (TIF) [file pone.0146588.s005.tif]

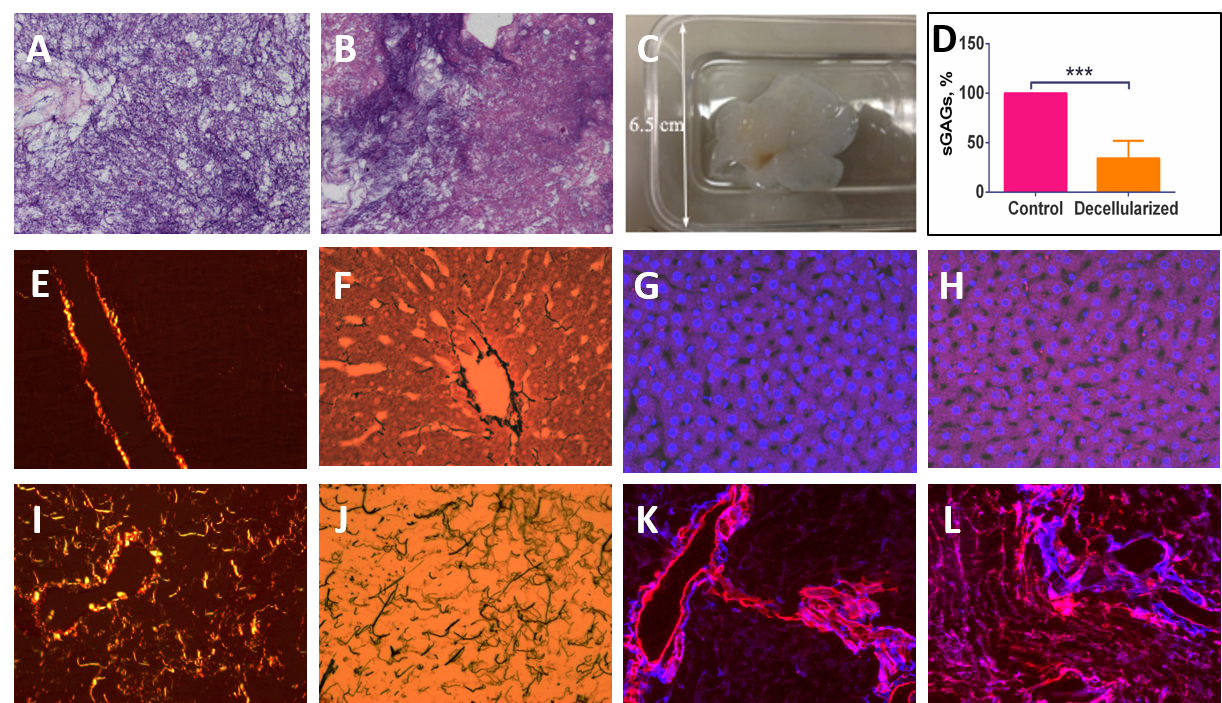

Supplement: S6 Fig — Livers were perfused in situ with increasing concentrations of Triton X-100 followed by 0.1% SDS (all in PBS), and were then washed with PBS. (A) Hematoxylin and eosin staining of normal decellularized liver. (B) Hematoxylin and eosin staining of liver treated for 2 weeks with CCl4 then decellularized. (C) Appearance of a decellularized normal liver. The size of the liver was maintained after perfusion. (D) Sulfated glycosaminoglycan (sGAG) content. Liver samples were analyzed with the Blyscan reagent. Livers decellularized by detergent perfusion retain on average 34% of initial GAGs. Mean +/- SD (normalized to 100% for control livers) of data from 3 individual livers. ***, p<0.0001 by t-test. (E-L) Paraffin sections of intact (E-H) and decellularized (I-L) normal livers stained with Sirius red (detects overall collagen; (E,I) and reticulin stains (collagen type III and proteoglycans; F, J) and with antibodies against laminin (G, K) and fibronectin (H, L). Collagen fibrils appear yellow when stained with sirius red and visualized with polarized light (E, I), and black when reticulin stained (F, J); for (G, H, K, L), specific stain is red, DAPI blue. Note the absence of specific staining for nuclei in the decellularized livers. (E, I) 40X; all others, 200X. (TIF) [file pone.0146588.s006.tif]
