# Supplementary material for: Normal and Fibrotic Rat Livers Demonstrate Shear Strain Softening and Compression Stiffening: A Model for Soft Tissue Mechanics
Source: PLoS One. 2016 Jan 6;11(1):e0146588. doi: 10.1371/journal.pone.0146588 (PMC4703410; doi:10.1371/journal.pone.0146588)
Supplement: S1 File — (A) Sample dimension parameters. (B) Predicted stress vs. strain λ (left) and modulus vs. strain λ (right), both normalized to C1. (C) Relation between G’ and shear strain γ. G’ decreases with application of shear strain. (D) Relation between E and axial strain λ. E increases with application of compressive strains and decreases with tension. (E) The model predictions for the relationship between G’ and axial strain λ. G’ increases with increasing compression and decreases with increasing tension, which is comparable to experimental data. (F) The model predictions compared with experimental data for the relationship between G’ and shear strain γ at different levels of compressive strains. G’ decreases with increasing shear at all levels of compression. (DOCX) [file pone.0146588.s007.docx]

**S1 Appendix: A non-linear constitutive model for the liver**

We present a minimal material model to simultaneously capture compression stiffening, tension softening, and shear softening behavior of liver. We write the strain energy density as ${W(\lambda}_{i})$, where $\lambda_{i}$ are stretches in the principle direction of the left-Green deformation tensor, $\boldsymbol{B}$. Using ${W(\lambda}_{i})$, we can determine the relation between principle stretch and principle stress:

$$\sigma_{i}\boldsymbol{(}\lambda_{j}\boldsymbol{)=}\frac{\partial W}{\partial\lambda_{i}}$$

Since the material is isotropic, $\sigma_{i}\boldsymbol{(}\lambda_{j}\boldsymbol{)}$ is symmetric under the rotation of index *i*. Let $l$ and $L$ be reference and current height of the sample respectively, $\theta$ be the torsion angle and $R$ be the sample radius (as shown in Figure A in S1 Appendix).


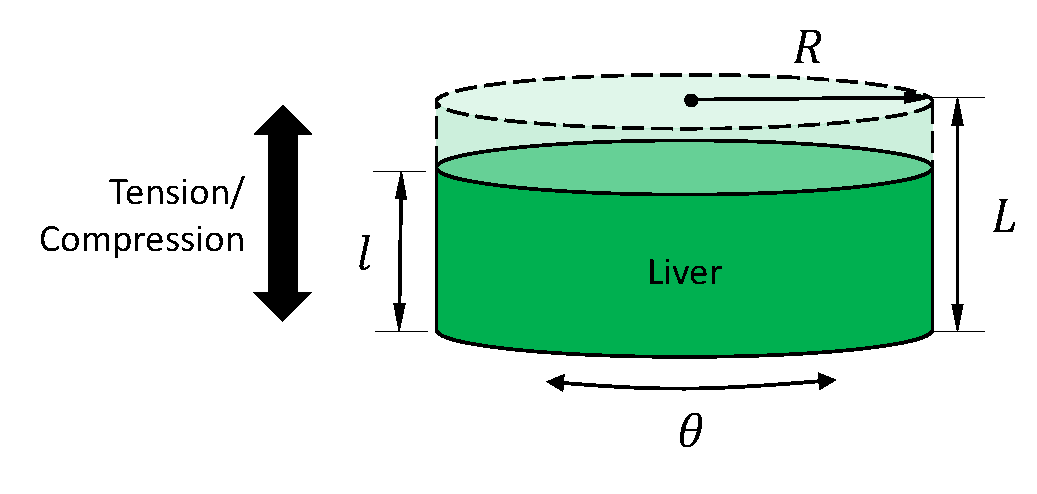


**Fig A. Sample dimension parameters.**

The uniaxial stretch ratio is given by $\lambda=l/L$ and the shear strain is $\gamma=\theta R/L$. The strain tensor is

$$\boldsymbol{B=}\left[ \begin{matrix} 1 & 0 & 0 \\ 0 & 1+\gamma^{2} & \gamma\lambda\\ 0 & \gamma\lambda& \lambda^{2} \end{matrix} \right]$$

and the 3 principle strains are:

$$\lambda_{1}=1$$

$$\lambda_{2}=\sqrt{\frac{1}{2}\left( 1+\lambda^{2}+\gamma^{2}+\sqrt{\left( 1+\lambda^{2}+\gamma^{2} \right)^{2}-4\lambda^{2}} \right)}$$

$$\lambda_{3}=\sqrt{\frac{1}{2}\left( 1+\lambda^{2}+\gamma^{2}-\sqrt{\left( 1+\lambda^{2}+\gamma^{2} \right)^{2}-4\lambda^{2}} \right)},$$

from which we can obtain principle stress:

$\boldsymbol{\sigma}_{\boldsymbol{i}}^{\boldsymbol{P}}\boldsymbol{=}\sigma_{i}\boldsymbol{(}\lambda_{j}\boldsymbol{)}$**.**

The stress in then given by $\boldsymbol{\sigma=Q\cdot}\boldsymbol{\sigma}^{\boldsymbol{P}}\boldsymbol{\cdot}\boldsymbol{Q}^{\boldsymbol{-1}}$, where $\boldsymbol{Q=}\sum_{\boldsymbol{i=1}}^{\boldsymbol{3}} \boldsymbol{n}_{\boldsymbol{i}}\boldsymbol{\bigotimes}\boldsymbol{e}_{\boldsymbol{i}}$ is the rotation matrix. Axes of $E$ and $G'$ measured in experiments are simply:

$$E=\frac{d\boldsymbol{\sigma}_{33}}{d\lambda}$$

$$G'=\frac{d\boldsymbol{\sigma}_{23}}{d\gamma}$$

Since $\boldsymbol{\sigma}_{\boldsymbol{23}}$, $\boldsymbol{\sigma}_{\boldsymbol{33}}$ are functions of $\sigma_{i}\boldsymbol{(}\lambda_{j}\boldsymbol{)}$, **the goal is to determine the scalar function** $\sigma_{i}\boldsymbol{(}\lambda_{j}\boldsymbol{)}$ using our experimental data.

We started with the case for shear with zero compression ($\lambda=1$, $\gamma\neq0$), for this case,

$$\lambda_{1}=1$$

$$\lambda_{2}=\sqrt{1+(\sqrt{\gamma^{2}\left( 4+\gamma^{2} \right)}+\gamma^{2})/2}$$

$\lambda_{3}=\sqrt{1-(\sqrt{\gamma^{2}\left( 4+\gamma^{2} \right)}-\gamma^{2})/2}$.

$\lambda_{2}>1$ (tension) and $\lambda_{3}<1$ (compression) and the shear stress is:

$$\tau\left( \gamma\right)=\frac{1}{\sqrt{\gamma^{2}+4}}(\sigma_{i}\left( \lambda_{2} \right)-\sigma_{i}(\lambda_{3}))$$

Since the principal tensile and compressive strains monotonically increase with shear strains, the material should soften in both compression and tension in order to capture the significant shear softening effect. Therefore, we designed the function form for stress with such properties using a simple power-law-function,

$$\sigma_{i}\left( \lambda_{i} \right)=\left\{ \begin{aligned} C_{1}\left( 1-\frac{1}{\lambda_{i}^{19}} \right), \lambda_{i}\geq1 \\ \frac{19C_{1}}{5}\left( \lambda_{i}^{5}-1 \right), \lambda_{i}<1 \end{aligned} \right.$$

The stress-strain and modulus-strain relationship of this material are shown in Figure B in S1 Appendix.


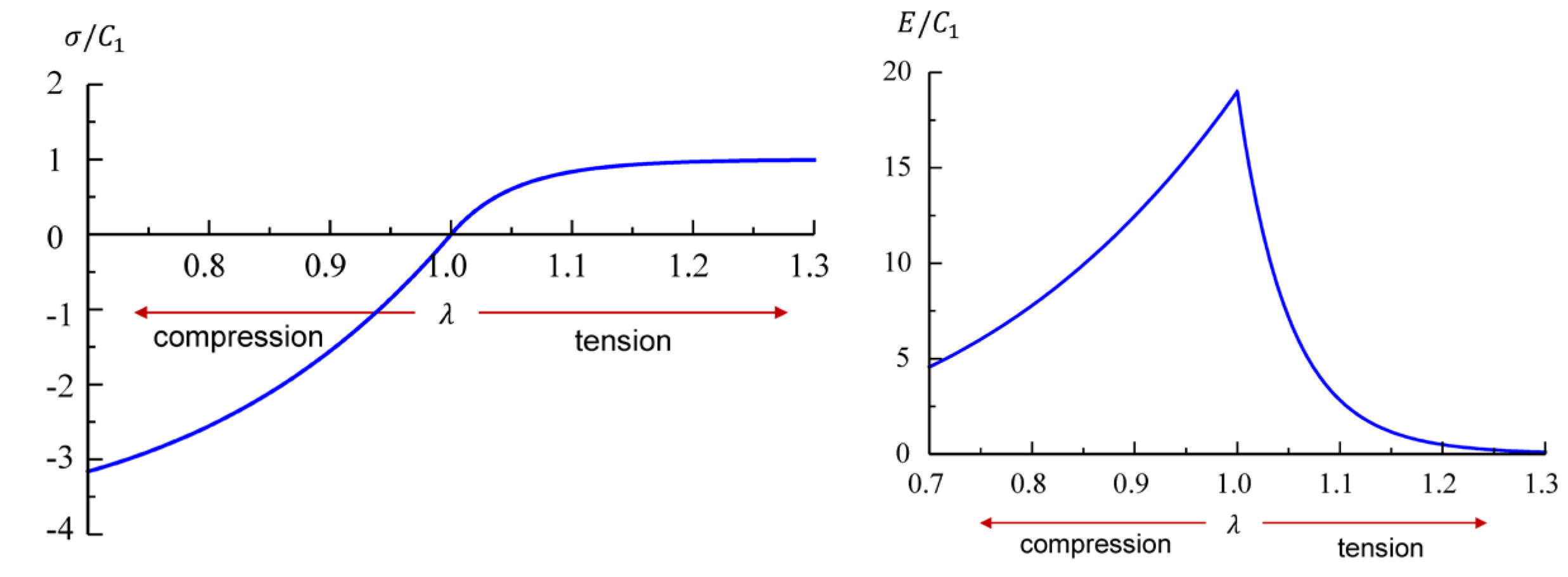


**Fig B. Predicted stress vs. strain λ (left) and modulus vs. strain λ (right), both normalized to** $\boldsymbol{C}_{\boldsymbol{1}}$**.**

However, since this model predicts compression softening, this functional form cannot explain the non-linear behavior of the Young’s modulus observed in our experiments. This is because the compressibility of the solid phase (which plays a role in uniaxial tension/compression tests, but not shear) has not been taken into consideration. The liver contains a high volume of water, which can flow in and out under mechanical load. While the liver can be considered incompressible overall, the solid phase can be compressible (as in the case of other biological tissues). Denoting the volume change by $=\sqrt{\det(\boldsymbol{B})}$, we modify the material model to

$$\sigma_{i}\left( \lambda_{i}, J \right)=\left\{ \begin{aligned} C_{1}\left( 1-\frac{1}{\lambda_{i}^{19}} \right), \lambda_{i}\geq1 \\ \frac{{19C}_{1}}{5J^{5}}\left( \lambda_{i}^{5}-1 \right),\lambda_{i}<1 \end{aligned}+C_{2}(1-\frac{1}{J^{9}}) \right.$$

The first factor accounts for the contribution primarily from deviatoric strains, and the second factor accounts for the contribution mainly from volumetric variations. The model is only characterized by 2 fitting parameters, $C_{1}\mathrm{and}C_{2}.$

***Fitting parameters and validation of the model predictions***

Starting with the shear softening data at zero compression ($J=1$), we obtain $C_{1}=55$ using the mean value of G’ from shear strain sweep experiments of 3 liver samples (Figure C in S1 Appendix). Next, we determine the parameter $C_{2}=100$by fitting the compression stiffening/tension softening data for E using the mean value (Figure D in S1 Appendix).


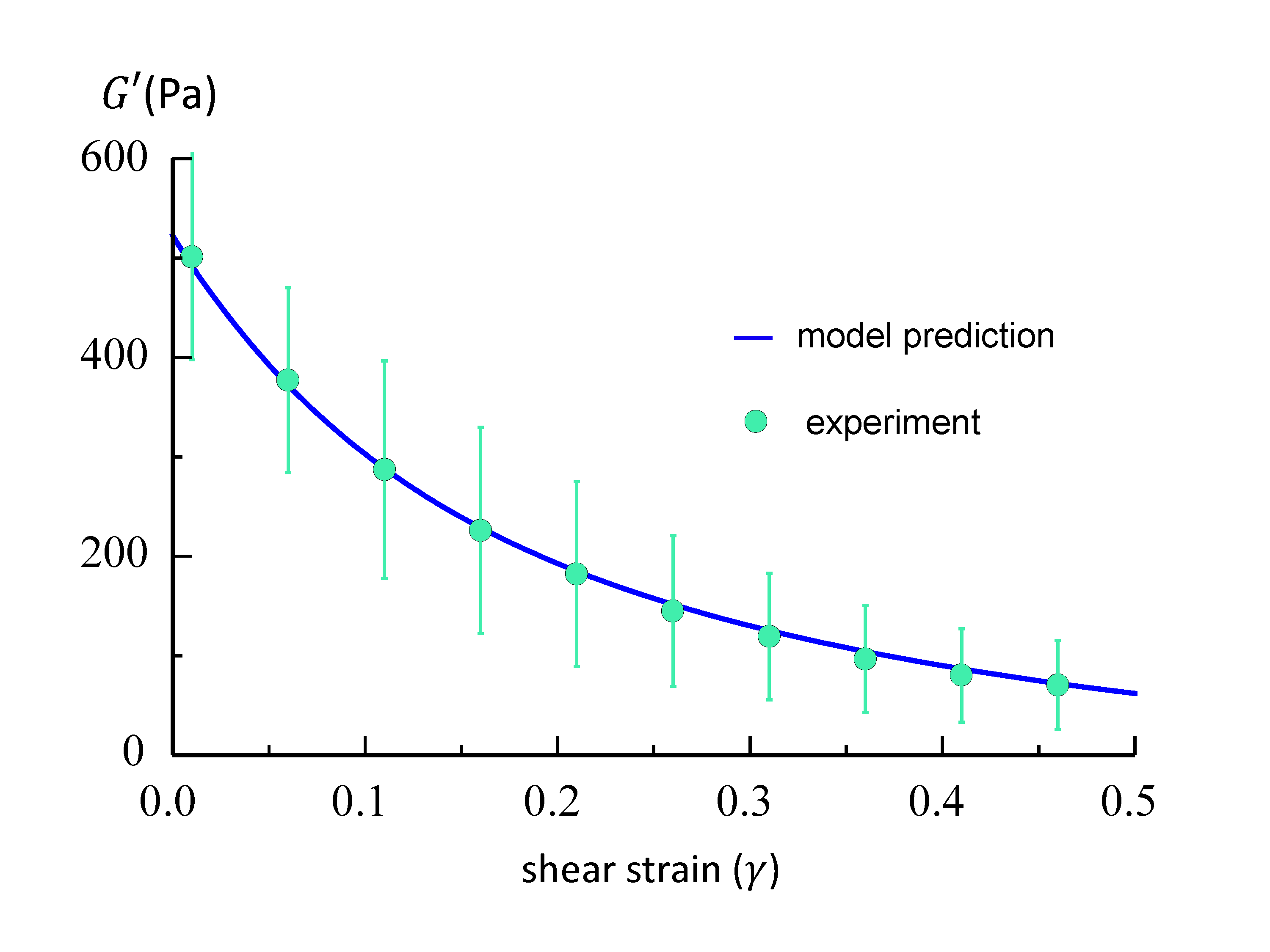


**Fig C. Relation between G’ and shear strain γ.** G’ decreases with application of shear strain.


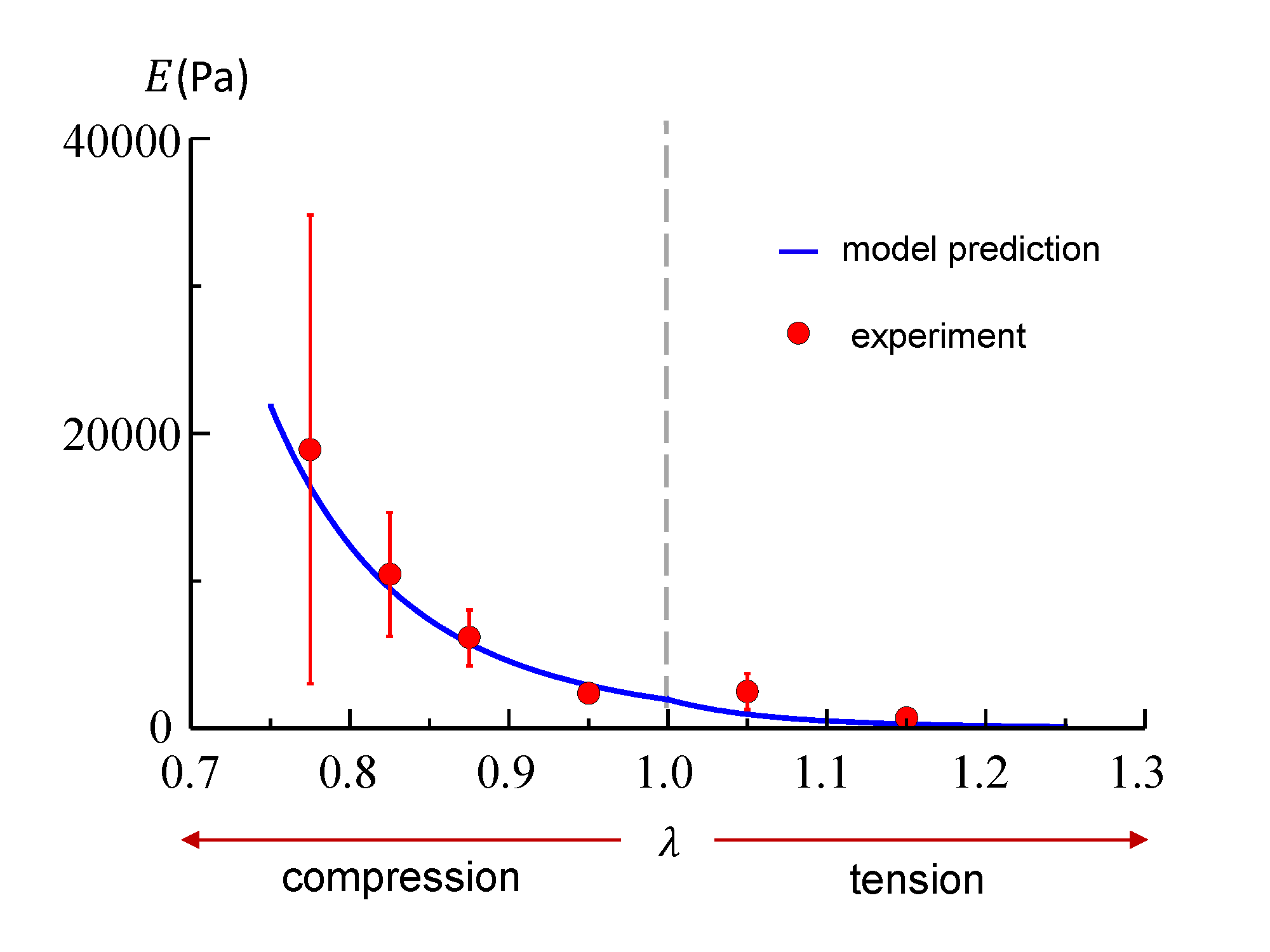


**Fig D. Relation between E and axial strain λ.** E increases with application of compressive strains and decreases with tension.

To validate the model, predicted values for G’ measured at 2% shear strain as a function of axial strain (Figure E in S1 Appendix) and G’ as function of shear strain at different levels of compressive strains (Figure F in S1 Appendix) are compared to the experimental data. The model predictions are in good agreement with the experimental results, which gives us confidence that the model captures the major elastic response of liver. To the best of our knowledge, this is the first model that captures both compression stiffening/tension softening and shear softening with only two material parameters, which satisfy,

$$E_{0}=\frac{d\sigma_{i}\left( \lambda_{i}, J \right)}{d\lambda_{i}}(\lambda_{i}=1)=19C_{1}+9C_{2}$$

where $E_{0}$ is the Young’s modulus with no compression/tension, $19C_{1}$ accounts for the contribution primarily from deviatoric strains, and $9C_{2}$ for the contribution mainly from volumetric variations.


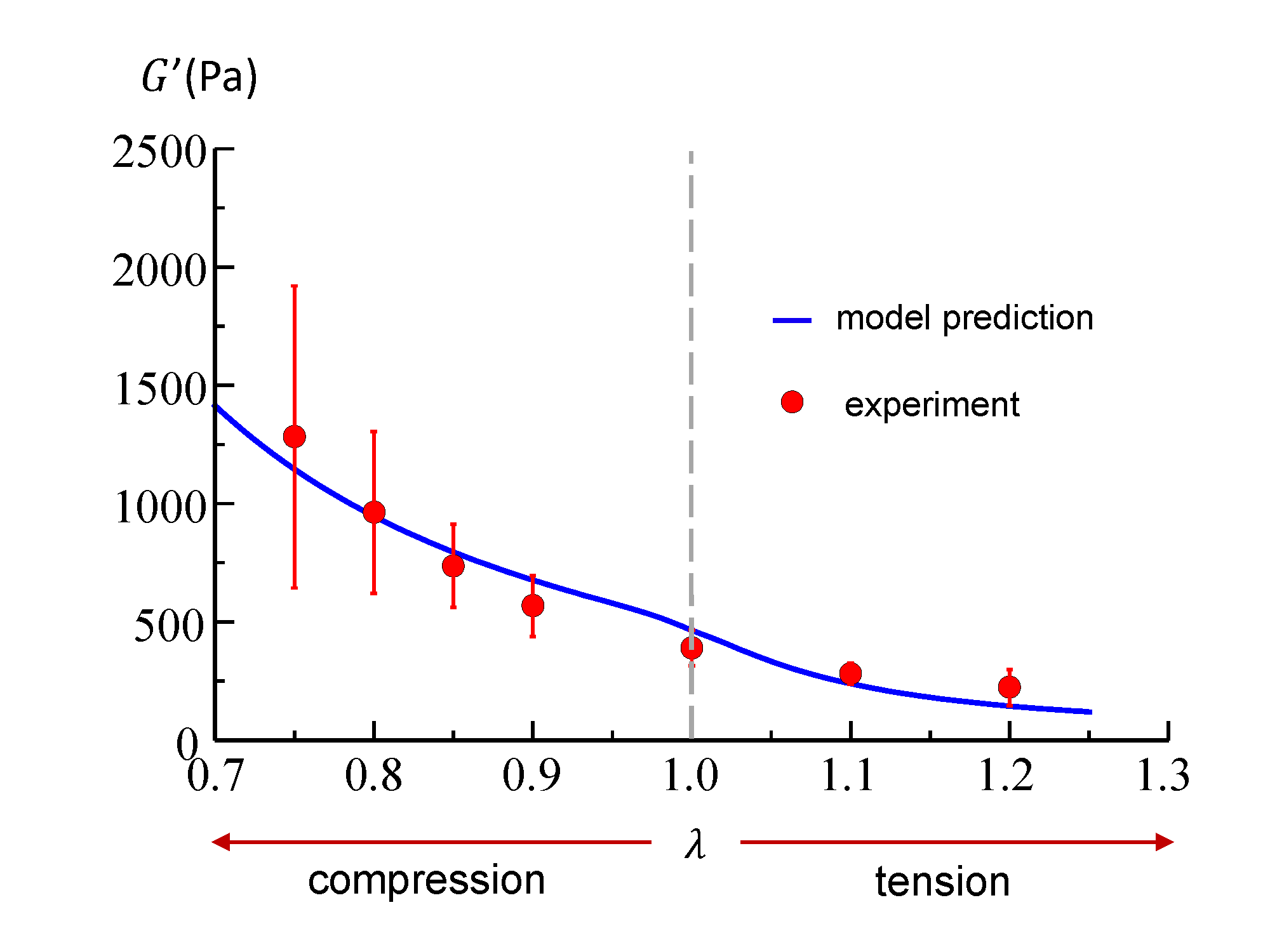


**Fig E. The model predictions for the relationship between G’ and axial strain λ.** G’ increases with increasing compression and decreases with increasing tension, which is comparable to experimental data.


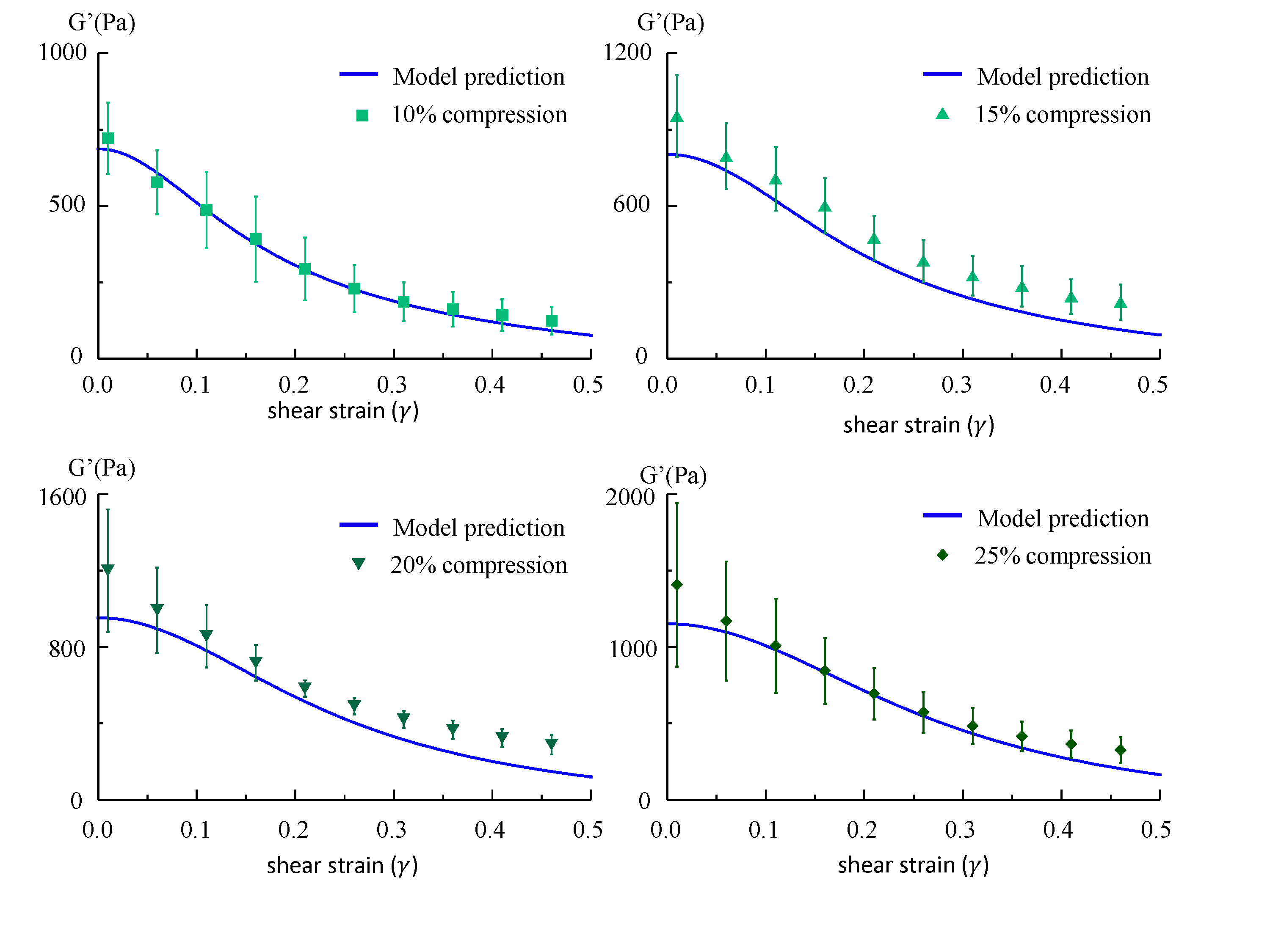


**Fig F. The model predictions compared with experimental data for the relationship between G’ and shear strain γ at different levels of compressive strains.** G’ decreases with increasing shear at all levels of compression.
